# P04.55. Examination of a staff massage break at a safety net hospital

**DOI:** 10.1186/1472-6882-12-S1-P325

**Published:** 2012-06-12

**Authors:** P Gardiner

**Affiliations:** 1 Boston Medical Center, Boston, USA

## Purpose

Workplace stress has been linked with health consequences and poor work performance. “Burnout” or mental exhaustion can lead to health conditions including impaired sleep and depression. Health care providers are especially prone to workplace stress. Therapeutic programs, like massage, in the workplace can be influential in supporting workers exposed to high levels of physical and mental stress.

## Methods

From 2010 to 2011, massage sessions were advertised through the nurse managers at Boston Medical Center (BMC), word of mouth, and signs for "Massage Break" posted every week there was a session. Participants were asked to fill out an evaluation form about the effect their massage had on them before and after their session. We used descriptive statistics and Student’s t tests.

## Results

BMC staff completed 313 massage evaluation forms. Forty-five percent (n=140) of participants were nurses. Other job types included, but were not limited to, physicians (10%, n=30) and social workers (6%, n=20). Number of times receiving a massage ranged from one to sixteen times. Responses about how the session helped ranged extensively. Eighteen percent of respondents (n=55) had comments related to improved work performance. Ten percent (n=30) of staff members liked that they felt cared for during the sessions and felt by taking the time to attend the session that they were practicing self care. There was a significant difference between before and after scores with regard to anxiety (t= - 3.56, p = 0.0004), stress (t= -3.36, p=0.0009), and pain (t= -3.58, t=0.0004).

## Conclusion

It is important for workplaces to support staff members and include opportunities for wellness during the workday. The benefits of massage can improve both the health and morale of the staff in this hospital community, especially for those delivering care to patients. More research is needed on incorporation of massage breaks into the workday for employees.

